# Association of *PDE4B* Polymorphisms with Susceptibility to Schizophrenia: A Meta-Analysis of Case-Control Studies

**DOI:** 10.1371/journal.pone.0147092

**Published:** 2016-01-12

**Authors:** Yanguo Feng, Dejun Cheng, Chaofeng Zhang, Yuchun Li, Zhiying Zhang, Juan Wang, Yuzhong Shi

**Affiliations:** Department of Psychiatry, Henan Mental Hospital, The Second Affiliated Hospital of Xinxiang Medical University, Xinxiang, China; Sudbury Regional Hospital, CANADA

## Abstract

**Background:**

The *PDE4B* single nucleotide polymorphisms (SNPs) have been reported to be associated with schizophrenia risk. However, current findings are ambiguous or even conflicting. To better facilitate the understanding the genetic role played by *PDE4B* in susceptibility to schizophrenia, we collected currently available data and conducted this meta-analysis.

**Methods:**

A comprehensive electronic literature searching of PubMed, Embase, Web of Science and Cochrane Library was performed. The association between *PDE4B* SNPs and schizophrenia was evaluated by odds ratios (ORs) and 95% confidence intervals (CIs) under allelic, dominant and recessive genetic models. The random effects model was utilized when high between-study heterogeneity (*I*^*2*^ > 50%) existed, otherwise the fixed effects model was used.

**Results:**

Five studies comprising 2376 schizophrenia patients and 3093 controls were finally included for meta-analysis. The rs1040716 was statistically significantly associated with schizophrenia risk in Asian and Caucasian populations under dominant model (OR = 0.87, 95% CI: 0.76–0.99, *P* = 0.04). The rs2180335 was significantly related with schizophrenia risk in Asian populations under allelic (OR = 0.82, 95% CI: 0.72–0.93, *P* = 0.003) and dominant (OR = 0.75, 95% CI: 0.64–0.88, *P* < 0.001) models. A significant association was also observed between rs4320761 and schizophrenia in Asian populations under allelic model (OR = 0.87, 95% CI: 0.75–1.00, *P* = 0.048). In addition, a strong association tendency was found between rs6588190 and schizophrenia in Asian populations under allelic model (OR = 0.87, 95% CI: 0.76–1.00, *P* = 0.055).

**Conclusion:**

This meta-analysis suggests that *PDE4B* SNPs are genetically associated with susceptibility to schizophrenia. However, due to limited sample size, more large-scale, multi-racial association studies are needed to further clarify the genetic association between various *PDE4B* variants and schizophrenia.

## Introduction

Susceptibility to schizophrenia has been considered to be inextricably related to genetic factors [1–4]. Twin studies have yielded heritability estimates of over 80% for schizophrenia, providing important genetic evidence for schizophrenia pathogenesis [5, 6]. In particular, to pinpoint genetic vulnerability to schizophrenia, genome-wide association studies have identified a number of single nucleotide polymorphisms (SNPs) within genes significantly associated with schizophrenia risk [7, 8].

Among various candidate genes for schizophrenia, the gene *phosphodiesterase 4B* (*PDE4B*), which is located on human chromosome 1 at 1p31 and composed of 17 exons spanning 580 kb [9], belongs to *PDE4* families. Like other adenosine 3’, 5’-monophosphate (cAMP) phosphodiesterases, PDE4B participates in cAMP signaling process by specifically inactivating cAMP [10]. Interestingly, mutations of fruit fly *dunce* gene, encoding an ortholog of mammalian PDE4, resulted in high levels of cAMP and deficiencies in olfactory learning and memory [11]. *PDE4B* was first implicated as a candidate risk gene for schizophrenia through investigating chromosomal abnormalities of two cousins, the proband of whom was diagnosed with schizophrenia [12]. Specifically, these cousins were both identified to carry a balanced t(1;16) (p31.2;q21) translocation and the locus encoding PDE4B was disrupted by the 1p31.2 translocation breakpoint [12]. In addition, disrupted in schizophrenia 1 (DISC1), another putative susceptibility factor for schizophrenia [13–17], was identified to interact with UCR2 domain of PDE4B and elevated cAMP contributed to decreased binding of PDE4B to DISC1 and an increase in PDE4B activity [12]. Therefore, it is highly probable that functional variants of PDE4B would affect multiple events, including interaction with DISC1, cAMP metabolism signaling and its cAMP hydrolyzing activity, with a concomitant complicated psychiatric outcome.

What is important, a genome-wide linkage analysis [18] in ethnically homogeneous pedigrees has provided strong evidence for schizophrenia risk locus on chromosome 1p31.1, to which the nearest schizophrenia candidate gene is *PDE4B*. Additionally, they identified 14 SNPs of *PDE4B* gene associated with schizophrenia under a nominal *P* value of 0.05 [18]. Indeed, there have been accumulating studies investigating association of *PDE4B* variations with predisposition to schizophrenia across multi-ethnic populations, including Caucasian populations (Europeans [19–21], Caucasian Canadians [22] and Caucasian Americans [23]), Asian populations (Indians [18], Japanese [24], Chinese [25] and Koreans [26]), and African populations (African Americans [23]). The only meta-analysis concerning the association of *PDE4B* SNPs and schizophrenia was taken in Bae’s study[26], which just combined Korean population in their own study and Japanese population in Numata’s study[24]. Another limitation of their meta-analysis is that it merely involves rs1040716 under dominant model and rs599381, rs2180335 and rs472952 under co-dominant model. Therefore, to better facilitate our interpretation of *PDE4B* SNPs as risk factors for schizophrenia and comprehensively evaluate the association between diverse SNPs and schizophrenia, we conducted this meta-analysis of published case-control studies across multi-ethnic populations under multi-genetic models.

## Methods and Materials

### Search strategy and Inclusion criteria

We conducted a comprehensive electronic literature searching of MEDLINE (PubMed), Embase (Ovid), Web of Science (Thomson-Reuters) and Cochrane Library (Wiley) from establishment date to August 2015. To maximize search scope and minimize the chance of missing relevant studies, we used a simple term combination strategy. The search terms and their combination manner were presented below: (“schizophrenia” OR “schizophrenic”) AND (“PDE4B” OR “phosphodiesterase 4B”). With this search strategy, there were 59, 86, 70, and 0 citations obtained from Pubmed, Embase, Web of Science and Cochrane Library, respectively. After excluding duplicates, there were 121 citations retrieved from these four electronic databases. In addition, to supplement the electronic search, we also manually searched reference lists of key studies and reviews for additional relevant studies, but no additional studies were obtained.

The eligible studies should satisfy the following criteria: (1) case-control studies; (2) written in English; (3) providing sufficient data to calculate the odds ratios (ORs) and 95% confidence intervals (CIs); (4) allele frequency and genotype distribution of control population must be in Hardy-Weinberg equilibrium; (5) stating that well-informed consent was acquired from all participants.

### Data extraction and quality assessment

For each included study, the following data were collected: the first author’s last name, publication year, country, ethnicity, numbers of cases and controls, diagnosis criteria, gender distribution, age and Hardy-Weinberg equilibrium. We calculated the ORs and 95%CIs, if they are not provided in original studies. Two researchers independently performed data extraction and discrepancy was resolved through discussion or referred to a third researcher.

The quality of the included studies was evaluated through a checklist originated from Strengthening the Reporting of Genetic Association (STREGA) recommendations for reports on genetic association studies [27] and modified according to the quality checklist depicted elsewhere [28].

### Meta-analysis

Heterogeneity between studies was evaluated by Cochran’s *Q* test and *I* squared (*I*^2^) statistics. If substantial heterogeneity was detected (*P* < 0.1 or *I*^2^ > 50%), the random effects model (the DerSimonian-Laird method) was employed; otherwise the fixed effects model (the Mantel-Haenszel method or the Inverse Variance method) was utilized. We assessed the association strength by using ORs and 95% CIs and the significance of pooled ORs was examined by *Z* test. To reflect the influence of a single study on between-study heterogeneity and the pooled effect sizes, we excluded one study each time and then observed the corresponding changes. To estimate potential publication bias, we performed trim and fill analysis [29]. The level of statistically significant differences was still considered at 0.05 without re-estimating significance thresholds, since the original data in this meta-analysis were not derived from high-throughput genome-wide association studies [30]. All analyses were conducted with Stata/SE 11.2 software (StataCorp., TX, USA).

## Results

### Study selection and characteristics of included studies

**Fig 1:**
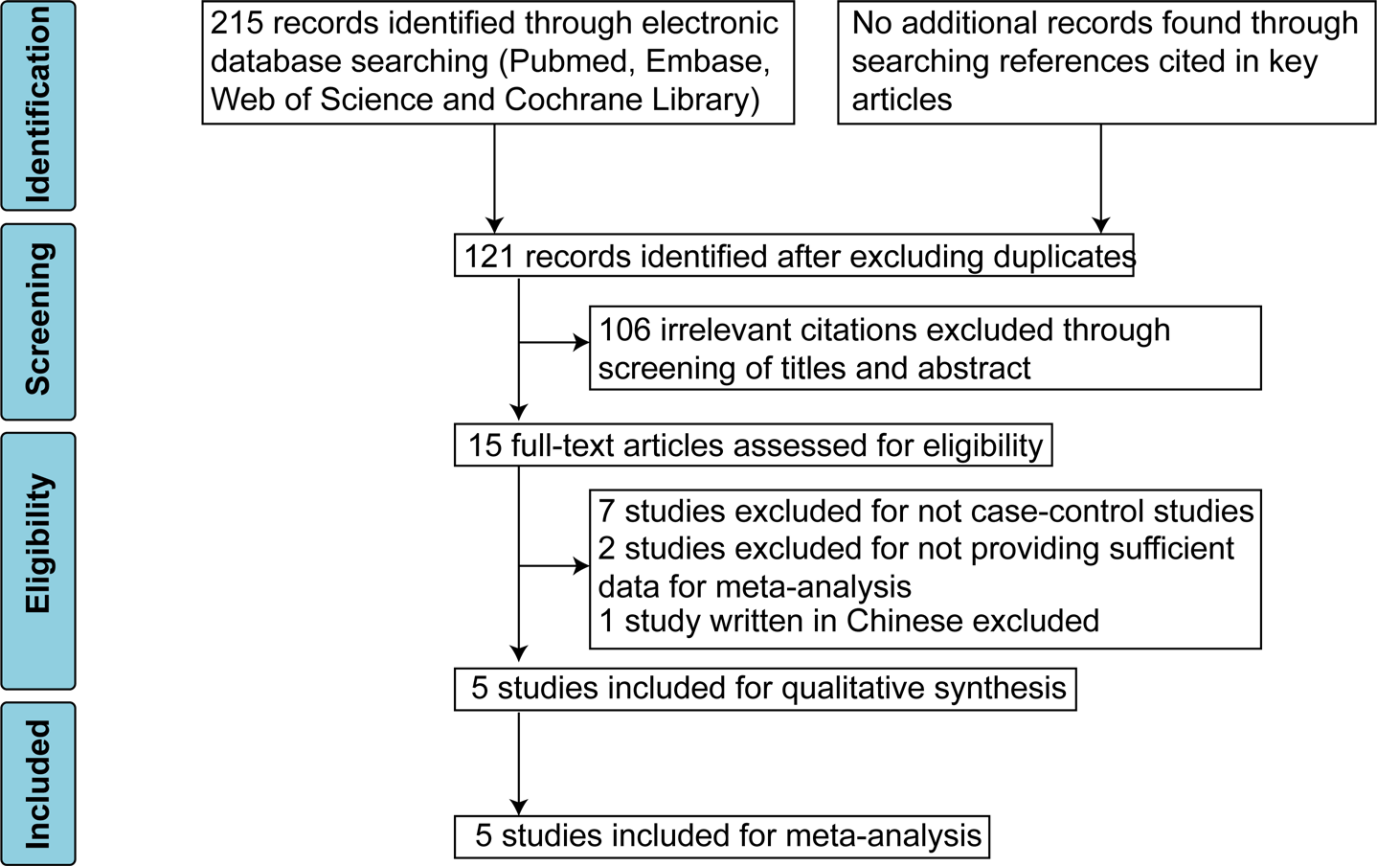
PRISMA flow diagram showing study selection process.

The PRISMA flow diagram of literature search was shown (Fig 1). In brief, after excluding duplicates, there were 121 citations retrieved. Next, 106 irrelevant citations were excluded through screening of titles and abstracts and 15 articles were assessed for eligibility. After excluding seven citations for being not case-control studies, two studies [19, 23] for insufficient data, though we tried to contact the authors for more information, and one study written in Chinese, five case-control studies [21, 22, 24–26] were finally included for this meta-analysis. The excluded articles and reasons were listed in S1 Text.

Main characteristics of the five included studies were described in Table 1. Briefly, two main ethnic populations, including Asians and Caucasians, from seven countries were involved in this study. In addition, *PDE4B* SNPs investigated in five studies are listed in Table 2. To appraise the quality of these included studies, a modified checklist was also provided in Table 3.

**Table 1:** Characteristics of included studies. M/F: male/female. SD: standard deviation. HWE: Hardy-Weinberg equilibrium. DSM-IV: Diagnostic Statistical Manual of Mental Disorders IV. ICD-10: International Classification of Diseases-10. NA: not available.

First author (publication year)	Country	Ethnicity	Sample size (Case/Control)	Diagnosis criteria	Gender (M/F)		Age (mean ± SD or range)		HWE
					Case	Control	Case (M/F)	Control (M/F)	
Numata (2008)	Japan	Asian	444/452	DSM-IV	265/179	271/181	48.4 ± 13.9/ 48.4 ± 15.0	48.7 ± 12.1/ 47.5 ± 12.7	yes
Guan (2012)	China	Asian	428/572	DSM-IV	226/202	298/274	38.4 ± 11.5/ 35.8 ± 10.9	31.3 ± 11.2/ 34.7 ± 11.3	yes
Bae (2015)	Korea	Asian	457/386	DSM-IV	255/202	217/169	44.78 (23–76)	54.72 (28–79)	yes
Kahler (2010)	Norway, Sweden, and Denmark	Caucasian	837/1473	DSM-IV/ ICD-10	489/348	848/625	NA	NA	yes
Rastogi (2009)	Canada	85% Caucasian, 7% African-American, 6% Asian, 1% East Indian and 1% other	210/210	DSM-IV	NA	NA	NA	NA	yes

**Table 2:** *PDE4B* SNPs investigated in each study. “+”: the SNP was investigated in the study. “-”: the SNP was not investigated in in the study.

	Numata (2008) Japanese	Guan (2012) Chinese	Bae (2015) Korean	Kahler (2010) Caucasian	Rastogi (2009) Mainly Caucasian
rs599381	+	+	+	+	-
rs1040716	+	+	+	+	-
rs472952	+	+	+	-	-
rs2180335	+	+	+	-	-
rs910694	+	+	-	-	+
rs4320761	+	+	-	-	-
rs498448	+	+	-	-	-
rs6588190	+	+	-	-	-

**Table 3:** Quality assessment of included studies. “+”: detailed description; “±”: incomplete description; “-”: no description.

Last name of first author	Year	Clear description of background, objectives and study design	Clear eligibility criteria	Clear definition of variables	Credible genotyping methods	Hardy-Weinberg equilibrium assessment	Clear description of statistical methods	Summary of characteristics of participants	Publicly available genotype data	Comprehensive discussion
Numata	2008	+	+	+	+	+	+	+	+	+
Rastogi	2009	+	+	+	+	+	+	±	+	+
Kahler	2010	+	+	+	+	+	+	±	+	+
Guan	2012	+	+	+	+	+	+	+	+	+
Bae	2015	+	+	+	+	+	+	+	±	+

### Location and potential function of SNPs in this meta-analysis

We searched for SNPs included into our meta-analysis at http://www.ncbi.nlm.nih.gov/snp/ and tabulated the information including locations and possible functions of these SNPs in Table 4.

**Table 4:** Location and potential function of SNPs in this meta-analysis.

SNP ID	Chromosome	Position	Intron Number	Functional Consequence
rs599381	1	66294877	Intron 7	Intron variant
rs1040716	1	66311907	Intron 7	Intron variant
rs472952	1	66335081	Intron 8	Intron variant
rs2180335	1	66320247	Intron 7	Intron variant
rs910694	1	66330543	Intron 7	Intron variant
rs4320761	1	66245285	Intron 3	Intron variant
rs498448	1	66301097	Intron 7	Intron variant
rs6588190	1	66231685	Intron 3	Intron variant

### Meta-analyses of association between different *PDE4B* SNPs and susceptibility to schizophrenia in allelic model

**Fig 2:**
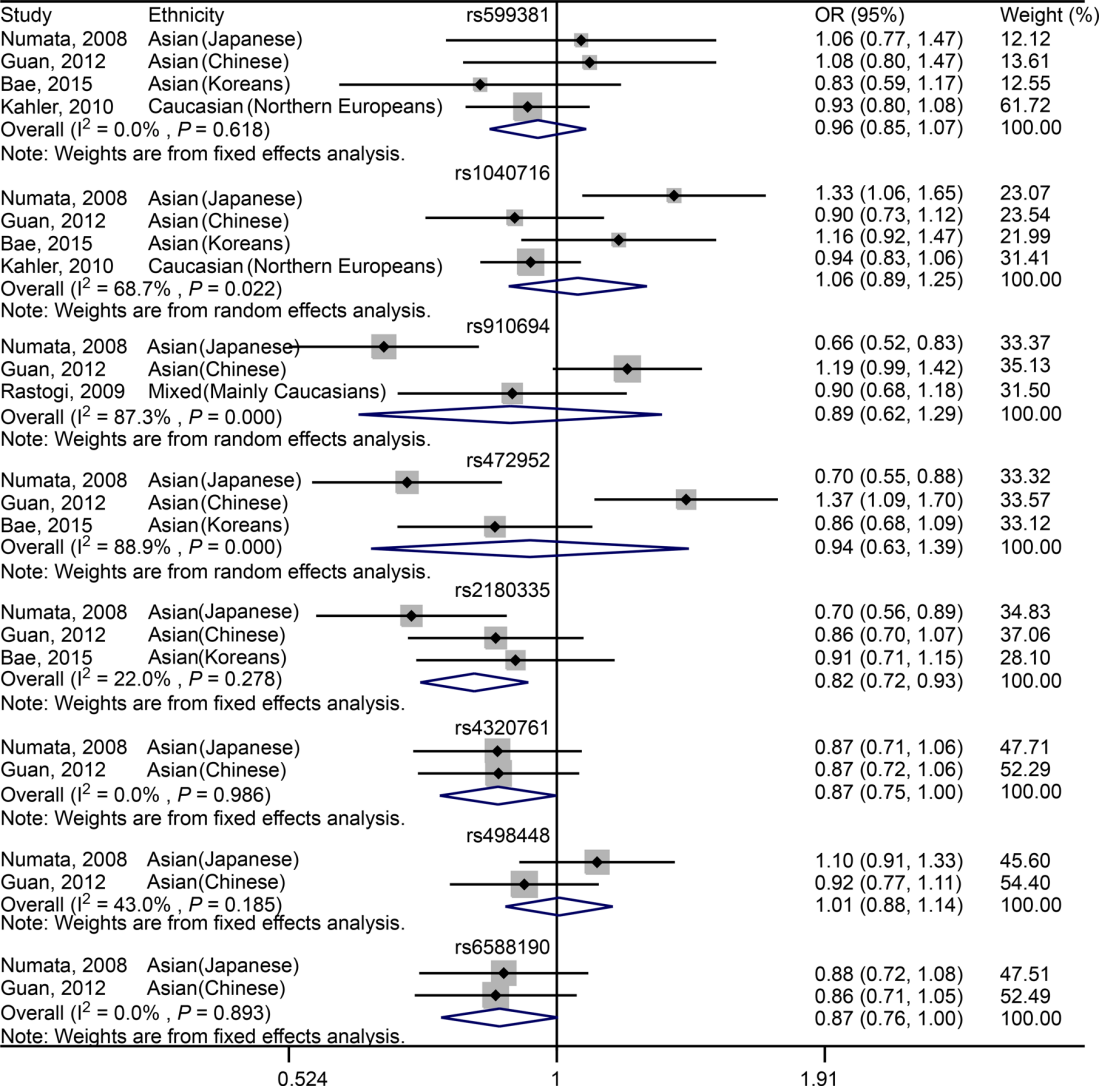
Forest plot showing the association between *PDE4B* SNPs and schizophrenia under allelic model.

For rs599381, no significant association was observed between this SNP and schizophrenia across Asian (including Japanese, Chinese and Koreans) and Caucasian (Northern Europeans) populations (OR = 0.96, 95% CI: 0.85–1.07, *P* = 0.443; Fig 2, Table 5). For Asian (including Japanese, Chinese and Koreans) subgroup analysis, no significant association was identified between rs599381 and schizophrenia (OR = 0.99, 95% CI: 0.82–1.20, *P* = 0.941; Table 5).

**Table 5:** Overall analysis of association of *PDE4B* SNP with schizophrenia risk.

SNP	Allele	Ethnicity	Cohort number	Case/Control	Genetic model	OR (95% CI)	*Z* score	*P*(*Z*)	*I*^*2*^ (%)
rs599381	C>T	Asian/Caucasian	4	2165/2881	T vs. C	0.96 (0.85, 1.07)	0.77	0.443	0
					TT+TC vs. CC	0.98 (0.86, 1.12)	0.31	0.759	0
					TT vs.TC+CC	0.92 (0.65, 1.31)	0.47	0.64	0
		Asian subgroup	3	1329/1409	T vs. C	0.99 (0.82, 1.20)	0.07	0.941	0
					TT+TC vs. CC	1.07 (0.87, 1.33)	0.66	0.507	0
					TT vs.TC+CC	0.90 (0.48, 1.67)	0.34	0.731	0
rs1040716	A>T	Asian/Caucasian	4	2164/2861	T vs. A	1.06 (0.89, 1.25)	0.62	0.537	68.7
					TT+TA vs. AA	0.87 (0.76, 0.99)	2.06	**0.04** *	0
					TT vs. TA+AA	1.07 (0.80, 1.43)	0.46	0.649	61.5
		Asian subgroup	3	1329/1407	T vs. A	1.11 (0.89, 1.40)	0.94	0.348	67.5
					TT+TA vs. AA	0.85 (0.71, 1.03)	1.68	**0.094 #**	3.6
					TT vs. TA+AA	1.11 (0.73, 1.69)	0.49	0.621	58.9
rs910694	A>G	Mixed population	3	1074/1225	G vs. A	0.89 (0.62, 1.29)	0.61	0.542	87.3
					GG+GA vs.AA	0.83 (0.51, 1.34)	0.76	0.448	85.9
					GG vs. GA+AA	1.10 (0.88, 1.38)	0.86	0.388	38.7
		Asian subgroup	2	872/1023	G vs. A	0.89 (0.50, 1.58)	0.41	0.685	93.6
					GG+GA vs.AA	0.81 (0.40, 1.63)	0.59	0.554	92.9
					GG vs. GA+AA	1.21 (0.93, 1.57)	1.44	0.150	24.3
rs472952	C>T	Asian	3	1329/1410	T vs. C	0.94 (0.63, 1.39)	0.32	0.747	88.9
					TT+TC vs. CC	0.85 (0.53, 1.36)	0.67	0.501	87.7
					TT vs. TC+CC	0.88 (0.41, 1.89)	0.32	0.749	78.4
rs2180335	C>T	Asian	3	1329/1410	T vs. C	0.82 (0.72, 0.93)	2.99	**0.003** *	22
					TT+TC vs. CC	0.75 (0.64, 0.88)	3.48	**< 0.001** *	47.3
					TT vs. TC+CC	0.81 (0.55, 1.18)	1.12	0.264	0
rs4320761	C>T	Asian	2	871/1024	T vs. C	0.87 (0.75, 1.00)	1.98	**0.048** *	0
rs498448	T>C	Asian	2	872/1024	C vs. T	1.01 (0.88, 1.14)	0.08	0.935	43
rs6588190	C>T	Asian	2	871/1024	T vs. C	0.87 (0.76, 1.00)	1.92	**0.055 #**	0

*, *P* < 0.05, showing statistically significant difference.

#, *P* < 0.1, showing strong association tendency.

For rs1040716, no significant association was found between this SNP and schizophrenia across Asian (including Japanese, Chinese and Koreans) and Caucasian (Northern Europeans) populations (OR = 1.06, 95% CI: 0.89–1.25, *P* = 0.537; Fig 2, Table 5). For Asian (including Japanese, Chinese and Koreans) subgroup analysis, no significant association was noticed between rs1040716 and schizophrenia (OR = 1.11, 95% CI: 0.89–1.40, *P* = 0.348; Table 5).

For rs910694, there is no significant association between rs910694 and schizophrenia in the mixed populations across the studies of Numata, Guan and Rastogi (OR = 0.89, 95% CI: 0.62–1.29, *P* = 0.542; Fig 2, Table 5). For Asian subgroup (including Japanese and Chinese) analysis, still no significant association was found (OR = 0.89, 95% CI: 0.50–1.58, *P* = 0.685; Table 5).

For rs472952, no significant association was determined between the SNP and schizophrenia within Asian populations, including Japanese, Chinese and Koreans (OR = 0.94, 95% CI: 0.63–1.39, *P* = 0.747; Fig 2, Table 5).

For rs2180335, a significant association was noted between the SNP and schizophrenia across Asian populations, including Japanese, Chinese and Koreans (OR = 0.82, 95% CI: 0.72–0.93, *P* = 0.003; Fig 2, Table 5).

For rs4320761, a significant association was seen between the SNP and schizophrenia across Asian populations, including Japanese and Chinese (OR = 0.87, 95% CI: 0.75–1.00, *P* = 0.048; Fig 2, Table 5).

For rs498448, no significant association was detected between the SNP and schizophrenia across Asian populations, including Japanese and Chinese (OR = 1.01, 95% CI: 0.88–1.14, *P* = 0.935; Fig 2, Table 5).

For rs6588190, a strong tendency rather than statistically significant association was revealed between the SNP and schizophrenia in Asian populations, including Japanese and Chinese (OR = 0.87, 95% CI: 0.76–1.00, *P* = 0.055; Fig 2, Table 5).

### Meta-analyses of association between different *PDE4B* SNPs and schizophrenia under dominant and recessive genetic models

**Fig 3:**
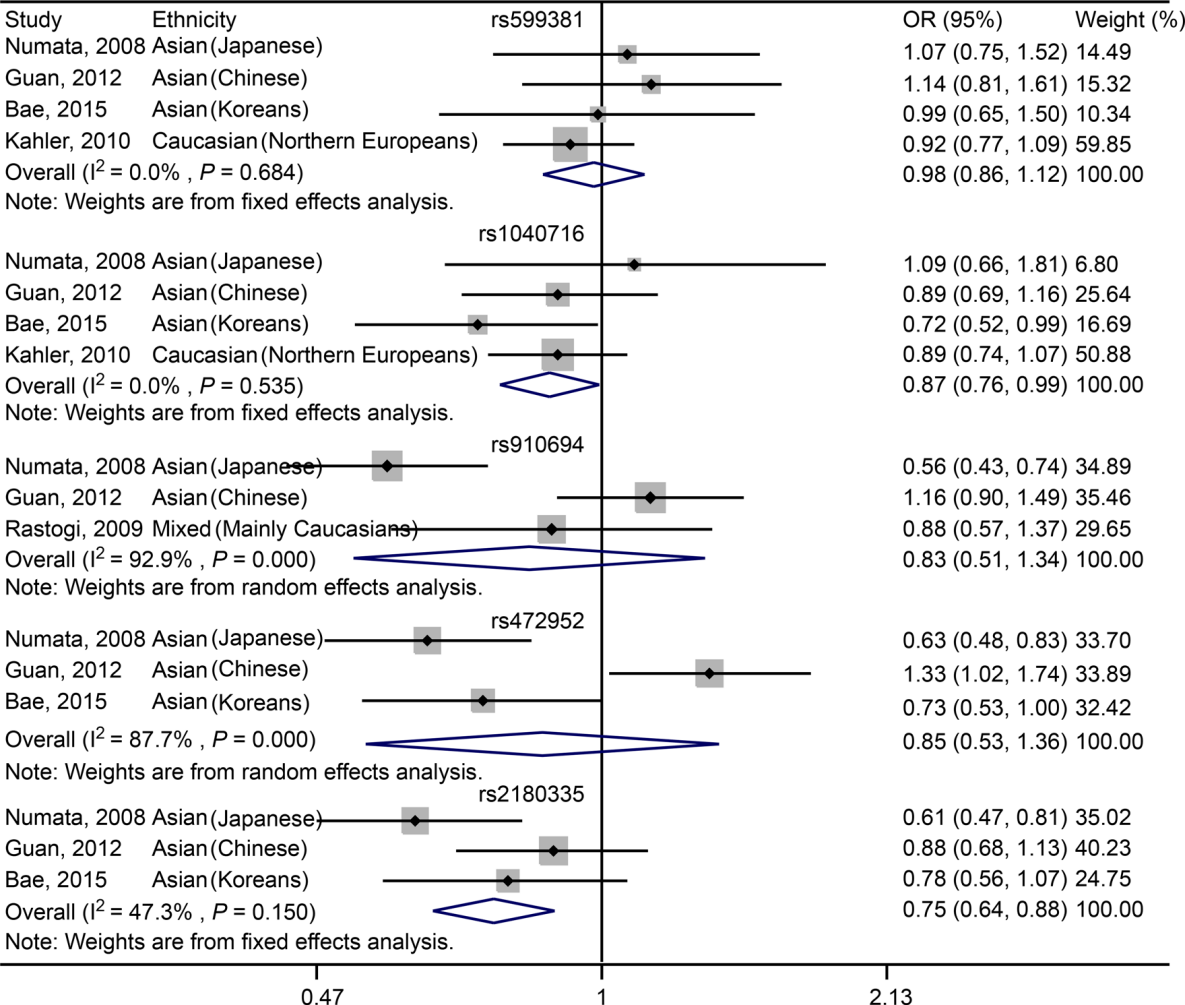
Forest plot showing the association between *PDE4B* SNPs and schizophrenia under dominant model.

**Fig 4:**
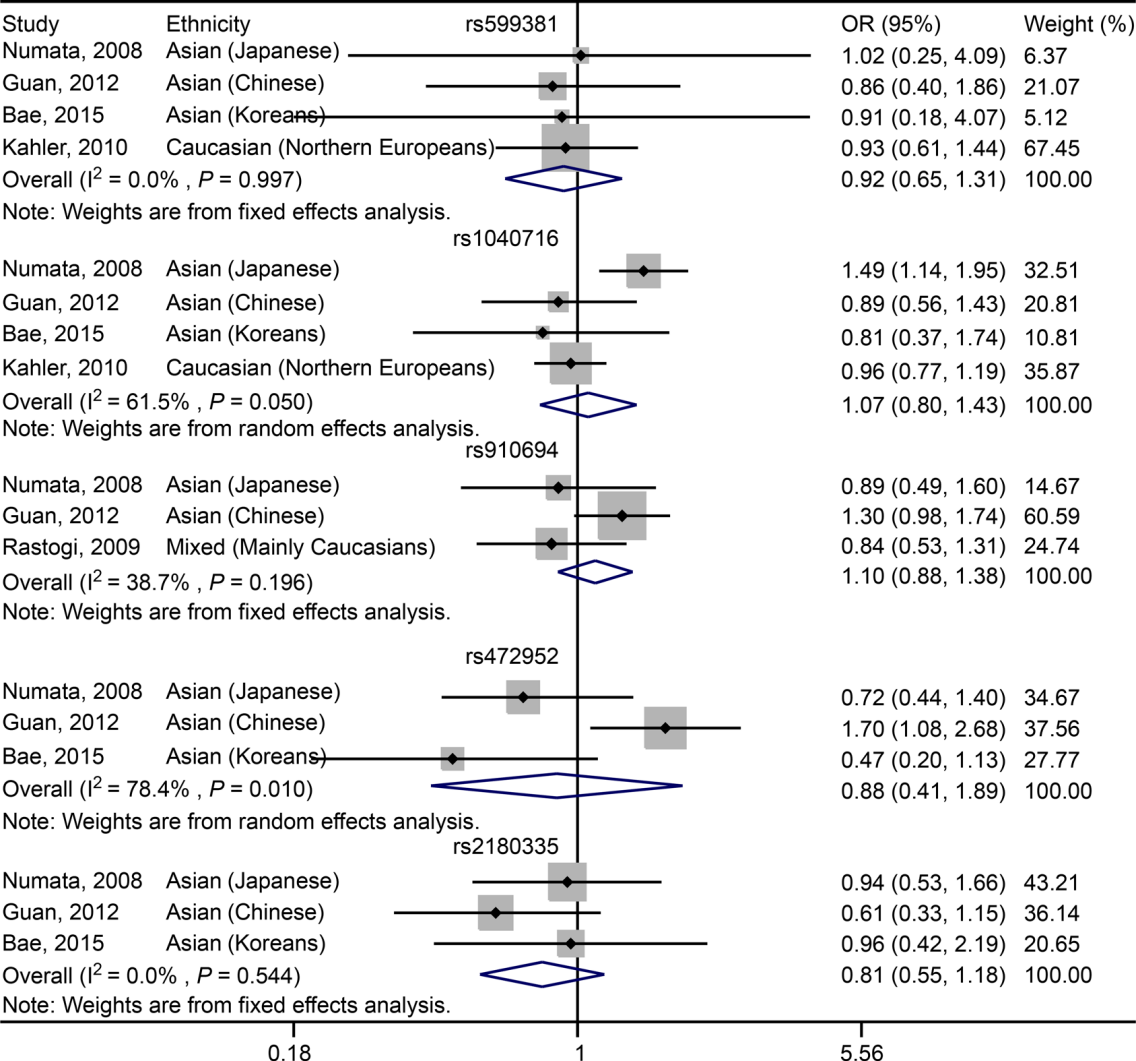
Forest plot showing the association between *PDE4B* SNPs and schizophrenia under recessive model.

For rs599381, no significant association was found between the SNP and susceptibility to schizophrenia in Asian (including Japanese, Chinese and Koreans) and Caucasian (Northern Europeans) populations under either dominant (OR = 0.98, 95% CI: 0.86–1.12, *P* = 0.759; Fig 3, Table 5) or recessive (OR = 0.92, 95% CI: 0.65–1.31, *P* = 0.64; Fig 4, Table 5) genetic model. For Asian (including Japanese, Chinese and Koreans) subgroup analysis, there is still no significant association between rs599381 and schizophrenia, in either dominant (OR = 1.07, 95% CI: 0.87–1.33, *P* = 0.507; Table 5) or recessive (OR = 0.90, 95% CI: 0.48–1.67, *P* = 0.731; Table 5) model.

For rs1040716, a significant association was characterized between the SNP and susceptibility to schizophrenia in Asian (including Japanese, Chinese and Koreans) and Caucasian (Northern Europeans) populations in dominant model (OR = 0.87, 95% CI: 0.76–0.99, *P* = 0.04; Fig 3, Table 5). However, under recessive model, there is no significant association was observed in Asian (including Japanese, Chinese and Koreans) and Caucasian (Northern Europeans) populations (OR = 1.07, 95% CI: 0.80–1.43, *P* = 0.649; Fig 4, Table 5). For Asian subgroup (including Japanese, Chinese and Koreans) analysis, no statistically significant association was found under dominant model (OR = 0.85, 95% CI: 0.71–1.03, *P* = 0.094; Table 5). Additionally, in recessive model, no significant association was characterized (OR = 1.11, 95% CI: 0.73–1.69, *P* = 0.621; Table 5).

For rs910694, no significant association was found between the SNP and schizophrenia in the Mixed populations across the studies of Numata, Guan and Rastogi in either dominant (OR = 0.83, 95% CI: 0.51–1.34, *P* = 0.448; Fig 3, Table 5) or recessive (OR = 1.10, 95% CI: 0.88–1.38, *P* = 0.388; Fig 4, Table 5) model. For Asian (including Japanese and Chinese) subgroup analysis, there is still no significant association was characterized in either dominant (OR = 0.81, 95% CI: 0.40–1.63, *P* = 0.55; Table 5) or recessive (OR = 1.21, 95% CI: 0.93–1.57, *P* = 0.15; Table 5) model.

For rs472952, no significant association was determined between the SNP and susceptibility to schizophrenia in Asian populations, including Japanese, Chinese and Koreans, in either dominant (OR = 0.85, 95% CI: 0.53–1.36, *P* = 0.501; Fig 3, Table 5) or recessive (OR = 0.88, 95% CI: 0.41–1.89, *P* = 0.749; Fig 4, Table 5) model.

For rs2180335, a significant association was detected between the variant and schizophrenia in Asian populations, including Japanese, Chinese and Koreans, under dominant genetic model (OR = 0.75, 95% CI: 0.64–0.88, *P* < 0.001; Fig 3, Table 5). However, under recessive model, no significant association was revealed (OR = 0.81, 95% CI: 0.55–1.18, *P* = 0.264; Fig 4, Table 5).

To conclude, the pooled analyses regarding association of *PDE4B* SNPs with schizophrenia under allelic, dominant and recessive models were summarized in Table 5.

### Sensitivity analysis

To test the influence of one individual study to the overall effect sizes, we performed sensitivity analysis of rs599381, rs1040716, rs472952 and rs2180335. For other SNPs, sensitivity analysis was not taken due to limited number of data sets. The results of sensitivity analysis were shown in Table 6.

**Table 6:** Sensitivity analysis of meta-analysis. The OR (95% CI) and *P* (*Z*) in this table are calculated when omitting the sensitive study.

SNP	Genetic model	Sensitive study	OR (95% CI)	*P* (*Z*)
rs599381	T vs. C	none		
	TT+TC vs. CC	none		
	TT vs.TC+CC	none		
rs1040716	T vs. A	none		
	TT+TA vs. AA	Guan, 2012	0.87 (0.74, 1.01)	0.061
		Bae, 2015	0.91 (0.78, 1.05)	0.174
		Kahler, 2010	0.85 (0.71, 1.03)	0.094
	TT vs. TA+AA	none		
rs910694	G vs. A	none		
	GG+GA vs. AA	none		
	GG vs. GA+AA	none		
rs472952	T vs. C	Guan, 2012	0.77 (0.63, 0.95)	0.015
	TT+TC vs. CC	Guan, 2012	0.67 (0.55, 0.83)	0.000
	TT vs. TC+CC	none		
rs2180335	T vs. C	Numata, 2008	0.88 (0.75, 1.03)	0.117
	TT+TC vs. CC	Numata, 2008	0.84 (0.69, 1.03)	0.088
	TT vs. TC+CC	none		

### Publication bias

To evaluate the potential publication bias in our meta-analysis, we merely employed trim and fill method [29] due to limited number of cohorts. For rs599381 in allelic model, trim and fill analysis showed that no potentially missing studies were found, suggesting that no publication bias existed. Under dominant model, trim and fill analysis revealed two potentially missing negative studies. Importantly, new meta-analysis after filling the two studies still showed no significant association between rs599381 and schizophrenia (*P* = 0.228). Furthermore, when under recessive model, trim and fill analysis found one possible missing study and new meta-analysis after filling this study still showed no significant association (*P* = 0.601). For rs1040716 under allelic model, using trim and fill analysis, no potentially missing study was found, suggesting no publication bias. In dominant model, trim and fill analysis discovered one potentially missing study. Of note, after filling this study, new meta-analysis still revealed a significant association between rs1040716 and schizophrenia risk (*P* = 0.017). In addition, trim and fill analysis predicted no missing study when meta-analysis on rs1040716 is under recessive model.

## Discussion

*PDE4B* was initially reported as a genetic susceptibility factor for schizophrenia in the exploration of chromosomal abnormalities of two psychiatric cousins [12]. Both cousins were identified with a balanced t(1;16) (p31.2;q21) translocation, which disrupted the *PDE4B* gene locus. Regarding the underlying mechanistic role played by PDE4B in the pathogenesis of schizophrenia, Millar et al. [12] demonstrated that DISC1, another established risk factor for schizophrenia, interacts with the UCR2 domain of PDE4B and increased level of cAMP gives rise to dissociation of PDE4B from DISC1 and an increase in PDE4B activity. In addition, it has been generally considered that the sole way to inactivate cAMP is through PDE action [10]. Therefore, it is reasonable to speculate that functional variants of PDE4B may lead to dysfunction of cAMP signaling and mediate complicated psychiatric outcome [31, 32].

Though there have been numerous association studies examining the genetic role of *PDE4B* in the etiology of schizophrenia, to date to our knowledge, no comprehensive meta-analysis was conducted to systematically summarize the association of *PDE4B* polymorphisms and schizophrenia. In order to better understand the genetic role of *PDE4B* for schizophrenia susceptibility, we recapitulated the association of different *PDE4B* SNPs with schizophrenia risk in multi-ethnic populations under allelic, dominant and recessive models.

For rs599381, no significant association was found between it and schizophrenia risk in Asian and Caucasian populations under allelic, dominant and recessive models. Sensitivity analysis revealed that this association was not influenced by any individual study. These analyses suggested that the polymorphism rs599381 may be not a risk SNP for schizophrenia, though more large-scale studies are still needed. For the association of rs1040716 with schizophrenia under allelic and recessive models, a large heterogeneity (Table 5) was observed. For Asian subgroup analysis, significant heterogeneity still existed in these two genetic models, indicating that ethnicity may be not the main cause for heterogeneity. However, under dominant model, no heterogeneity existed in Asian and Caucasian populations and a rather small heterogeneity (*I*^*2*^ = 3.6%) appeared in Asian populations. Sensitivity analysis demonstrated that the statistically significant association between rs1040716 and schizophrenia risk under dominant model was seemingly unstable and correspondingly changed with a certain study deleted (Table 6). On one hand, it is possible that the Bae’ study overestimated the pooled effect size. On the other hand, though statistically significant association disappeared when Guan’s or Kahler’s study was omitted, a careful scrutiny will find that a strong association tendency (*P* = 0.061, *P* = 0.094) still existed. In addition, the new meta-analysis of association between rs1040716 and schizophrenia under dominant model after filling one possible missing study detected by trim and fill analysis remained significant (*P* = 0.017). Therefore, this result should be interpreted with caution and more large-scale replicative studies are necessary to ascertain the relationship. For analysis of rs472952, high heterogeneity was observed in allelic, dominant and recessive genetic models in Asian populations, suggesting that ethnicity may be not responsible for high heterogeneity. In addition, sensitivity analysis showed that heterogeneity dramatically decreased when Guan’s study was removed in allelic, dominant, and recessive models (allelic model: *I*^*2*^ from 88.9% to 37.7%; dominant model: *I*^*2*^ from 87.7% to 0%; recessive model: *I*^*2*^ from 78.4% to 0%). We assume that this heterogeneity variation may be not coincidental because similar change was observed in three genetic models. It is highly probable that the significant heterogeneity between studies was attributable partly to geographic factors, age difference in samples (seen from Table 1), lifestyle diversity or sampling difference. For rs2180335, sensitivity analysis displayed that study from Numata may overestimate the pooled odds ratios (Table 6) and therefore the significant association under allelic and dominant model should be cautiously interpreted. As for rs910694, rs4320761, rs498448 and rs6588190, more large-scale replicative studies are required due to their limited cohort number and sample size.

Besides the studies included into our meta-analysis, there are some other studies demonstrating the genetic association between *PDE4B* SNPs and schizophrenia. Pickard et al. [19] identified that two *PDE4B* SNPs, rs2503177 and rs2503166, were significantly associated with schizophrenia in female Scottish case-control populations, conferring a protective effect against schizophrenia in females. Another case-control study [23] revealed that several SNPs within *PDE4B* gene including rs1354064, rs4320761, rs1040716, rs910694, rs1321177, rs2144719 and rs783038 were significantly associated with schizophrenia in Caucasian American population; in addition, rs599381, rs1040716 and rs910694 were shown to be significantly associated with schizophrenia in African American population. In addition, these significantly schizophrenia-associated SNPs are mostly located in introns adjacent to splice junctions, which indicates that genetic variants in introns surrounding critical splice junctions within the *PDE4B* gene are associated with increased incidence of schizophrenia [23]. Furthermore, a candidate gene association study based on Finnish pedigrees [20] identified that the *PDE4B* SNP rs7412571 was significantly associated with schizophrenia (*P* = 0.018). Two allelic haplotypes of *PDE4B* were observed in statistical significance (*P* = 0.0022 and *P* = 0.029), increasing or decreasing schizophrenia susceptibility [20].

To conclude, our meta-analysis suggest that rs1040716, rs2180335 and rs4320761 may serve as genetic susceptibility factors for schizophrenia. Moreover, a strong association tendency between rs6588190 and schizophrenia risk was found. However, due to limitations in our meta-analysis, more large-scale studies from different ethnic populations are needed to further ascertain the underlying relationship between *PDE4B* SNPs and predisposition to schizophrenia.

## Supporting Information

S1 Checklist PRISMA checklist. (DOC)

S2 Checklist Meta-analysis on genetic association studies form checklist. (DOCX)

S1 Text List of excluded citations and reasons. (DOCX)
