# Supplementary material for: Association of PDE4B Polymorphisms with Susceptibility to Schizophrenia: A Meta-Analysis of Case-Control Studies
Source: PLoS One. 2016 Jan 12;11(1):e0147092. doi: 10.1371/journal.pone.0147092 (PMC4710508; doi:10.1371/journal.pone.0147092)
Supplement: S1 Text — (DOCX) [file pone.0147092.s003.docx]

**1. 106 Citations excluded through screening of titles and abstracts.**

1. Zhang X, Li X, Li M, Ren J, Yun K, An Y, et al. Venlafaxine increases cell proliferation and regulates DISC1, PDE4B and NMDA receptor 2B expression in the hippocampus in chronic mild stress mice. European journal of pharmacology. 2015;755:58-65. Epub 2015/03/15. doi: 10.1016/j.ejphar.2015.02.044. PubMed PMID: 25769842.

2. Yang H, Murigi FN, Wang Z, Li J, Jin H, Tu Z. Synthesis and in vitro characterization of cinnoline and benzimidazole analogues as phosphodiesterase 10A inhibitors. Bioorganic & Medicinal Chemistry Letters. 2015;25(4):919-24. doi: 10.1016/j.bmcl.2014.12.054. PubMed PMID: WOS:000349720400034.

3. Salemi M, Condorelli RA, La Vignera S, Castiglione R, Salluzzo MG, Bonaccorso CM, et al. Expression of Phosphodiesterase 4B cAMP-Specific Gene in Subjects With Cryptorchidism and Down's Syndrome. Journal of clinical laboratory analysis. 2015.

4. Hedde J, Semproni A, Graf R, Schmidt CJ, Hughes ZA. The phosphodiesterase-4 inhibitor, ABI-4, attenuates the increases in brain cytokines and translocator protein binding caused by lipopolysaccharide. Schizophrenia Bulletin. 2015;41:S4-S5.

5. Dorner-Ciossek C, Giovannini R, Rosenbrock H. BI 409306, A novel phosphodiesterase 9A inhibitor, Part I: Potency, selectivity and in-vitro functional characterization on synaptic plasticity. Schizophrenia Bulletin. 2015;41:S31.

6. Chang EH, Kirtley A, Chandon TSS, Borger P, Husain-Krautter S, Vingtdeux V, et al. Postnatal neurodevelopmental expression and glutamate-dependent regulation of the ZNF804A rodent homologue. Schizophrenia research. 2015.

7. Xu W, Cohen-Woods S, Chen Q, Noor A, Knight J, Hosang G, et al. Genome-wide association study of bipolar disorder in Canadian and UK populations corroborates disease loci including SYNE1 and CSMD1. Bmc Medical Genetics. 2014;15. doi: 10.1186/1471-2350-15-2. PubMed PMID: WOS:000330068200001.

8. Sheppard CL, Lee LC, Hill EV, Henderson DJ, Anthony DF, Houslay DM, et al. Mitotic activation of the DISC1-inducible cyclic AMP phosphodiesterase-4D9 (PDE4D9), through multi-site phosphorylation, influences cell cycle progression. Cellular signalling. 2014;26(9):1958-74. Epub 2014/05/13. doi: 10.1016/j.cellsig.2014.04.023. PubMed PMID: 24815749.

9. Salemi M, Condorelli RA, La Vignera S, Castiglione R, Salluzzo MG, Bonaccorso CM, et al. Expression of Phosphodiesterase 4B cAMP-Specific Gene in Subjects With Cryptorchidism and Down's Syndrome. Journal of clinical laboratory analysis. 2014. Epub 2014/12/30. doi: 10.1002/jcla.21835. PubMed PMID: 25546171.

10. Babu PV, Gorja DR, Meda CLT, Deora GS, Kolli SK, Parsa KVL, et al. Synthesis of N-(3-arylprop-2-ynyl)substituted olanzapine derivatives as potential inhibitors of PDE4B. Tetrahedron Letters. 2014;55(20):3176-80. doi: 10.1016/j.tetlet.2014.04.009. PubMed PMID: WOS:000336700400010.

11. Azam MA, Tripuraneni NS. Selective Phosphodiesterase 4B Inhibitors: A Review. Scientia pharmaceutica. 2014;82(3):453-81. Epub 2015/04/09. doi: 10.3797/scipharm.1404-08. PubMed PMID: 25853062; PubMed Central PMCID: PMCPmc4318138.

12. Soda T, Frank C, Ishizuka K, Baccarella A, Park YU, Flood Z, et al. DISC1-ATF4 transcriptional repression complex: dual regulation of the cAMP-PDE4 cascade by DISC1. Molecular psychiatry. 2013;18(8):898-908. Epub 2013/04/17. doi: 10.1038/mp.2013.38. PubMed PMID: 23587879; PubMed Central PMCID: PMCPmc3730299.

13. Richter W, Menniti FS, Zhang HT, Conti M. PDE4 as a target for cognition enhancement. Expert Opinion on Therapeutic Targets. 2013;17(9):1011-27.

14. Paspalas CD, Wang M, Arnsten AF. Constellation of HCN channels and cAMP regulating proteins in dendritic spines of the primate prefrontal cortex: potential substrate for working memory deficits in schizophrenia. Cerebral cortex (New York, NY : 1991). 2013;23(7):1643-54. Epub 2012/06/14. doi: 10.1093/cercor/bhs152. PubMed PMID: 22693343; PubMed Central PMCID: PMCPmc3673177.

15. Miller BJ, Culpepper N, Rapaport MH, Buckley P. Prenatal inflammation and neurodevelopment in schizophrenia: A review of human studies. Progress in Neuro-Psychopharmacology and Biological Psychiatry. 2013;42:92-100.

16. Li J, Jin H, Zhou H, Rothfuss J, Tu Z. Synthesis and in vitro biological evaluation of pyrazole group-containing analogues for PDE10A. Medchemcomm. 2013;4(2):443-9. doi: 10.1039/c2md20239e. PubMed PMID: WOS:000314311600021.

17. Schmidt-Kastner R, Van Os J, Esquivel G, Steinbusch HWM, Rutten BPF. An environmental analysis of genes associated with schizophrenia: Hypoxia and vascular factors as interacting elements in the neurodevelopmental model. Molecular psychiatry. 2012;17(12):1194-205.

18. Lipina TV, Wang M, Liu F, Roder JC. Synergistic interactions between PDE4B and GSK-3: DISC1 mutant mice. Neuropharmacology. 2012;62(3):1252-62. Epub 2011/03/08. doi: 10.1016/j.neuropharm.2011.02.020. PubMed PMID: 21376063.

19. Kuroiwa M, Snyder GL, Shuto T, Fukuda A, Yanagawa Y, Benavides DR, et al. Phosphodiesterase 4 inhibition enhances the dopamine D1 receptor/PKA/DARPP-32 signaling cascade in frontal cortex. Psychopharmacology. 2012;219(4):1065-79. Epub 2011/08/13. doi: 10.1007/s00213-011-2436-8. PubMed PMID: 21833500; PubMed Central PMCID: PMCPmc3539205.

20. Johansson EM, Reyes-Irisarri E, Mengod G. Comparison of cAMP-specific phosphodiesterase mRNAs distribution in mouse and rat brain. Neuroscience letters. 2012;525(1):1-6. Epub 2012/08/14. doi: 10.1016/j.neulet.2012.07.050. PubMed PMID: 22884617.

21. Girgenti MJ, LoTurco JJ, Maher BJ. ZNF804a regulates expression of the schizophrenia-associated genes PRSS16, COMT, PDE4B, and DRD2. PloS one. 2012;7(2):e32404. Epub 2012/03/03. doi: 10.1371/journal.pone.0032404. PubMed PMID: 22384243; PubMed Central PMCID: PMCPmc3288100.

22. Clark S, Aberg K, Nerella S, Kumar G, McClay J, Xie L, et al. Using next generation sequencing to investigate methylation patterns associated with alcohol use behaviors. Behavior Genetics. 2012;42(6):915-6.

23. Burgin A. Structure based design of PDE4 allosteric modulators. Hormone and Metabolic Research. 2012;44(10).

24. Bradshaw NJ, Porteous DJ. DISC1-binding proteins in neural development, signalling and schizophrenia. Neuropharmacology. 2012;62(3):1230-41. Epub 2011/01/05. doi: 10.1016/j.neuropharm.2010.12.027. PubMed PMID: 21195721; PubMed Central PMCID: PMCPmc3275753.

25. Tropea D, Molinos I, Petit E, Baum M, Ajetnmobi A, Bellini S, et al. Circuit reorganization in disrupted in schizophrenia 1 (DISC1) mutant mice. Society for Neuroscience Abstract Viewer and Itinerary Planner. 2011;41. PubMed PMID: BIOSIS:PREV201200050312.

26. Soares DC, Carlyle BC, Bradshaw NJ, Porteous DJ. DISC1: Structure, Function, and Therapeutic Potential for Major Mental Illness. Acs Chemical Neuroscience. 2011;2(11):609-32. doi: 10.1021/cn200062k. PubMed PMID: WOS:000297143400001.

27. Petti M, Samanich J, Pan Q, Huang C-K, Reinmund J, Farooqi S, et al. Molecular Characterization of an Interstitial Deletion of 1p31.3 in a Patient With Obesity and Psychiatric Illness and a Review of the Literature. American Journal of Medical Genetics Part A. 2011;155A(4):825-32. doi: 10.1002/ajmg.a.33869. PubMed PMID: WOS:000288792700022.

28. Otowa T, Kawamura Y, Sugaya N, Yoshida E, Shimada T, Liu X, et al. Association study of PDE4B with panic disorder in the Japanese population. Progress in Neuro-Psychopharmacology & Biological Psychiatry. 2011;35(2):545-9. doi: 10.1016/j.pnpbp.2010.12.013. PubMed PMID: WOS:000289811200034.

29. O'Kane CJ. Drosophila as a model organism for the study of neuropsychiatric disorders. 2011. p. 37-60.

30. Newburn EN, Hyde TM, Ye T, Morita Y, Weinberger DR, Kleinman JE, et al. Interactions of human truncated DISC1 proteins: implications for schizophrenia. Translational psychiatry. 2011;1:e30. Epub 2011/01/01. doi: 10.1038/tp.2011.31. PubMed PMID: 22832604; PubMed Central PMCID: PMCPmc3309510.

31. MacKenzie KF, Wallace DA, Hill EV, Anthony DF, Henderson DJ, Houslay DM, et al. Phosphorylation of cAMP-specific PDE4A5 (phosphodiesterase-4A5) by MK2 (MAPKAPK2) attenuates its activation through protein kinase A phosphorylation. The Biochemical journal. 2011;435(3):755-69. Epub 2011/02/18. doi: 10.1042/bj20101184. PubMed PMID: 21323643.

32. Kvajo M, McKellar H, Drew LJ, Lepagnol-Bestel AM, Xiao L, Levy RJ, et al. Altered axonal targeting and short-term plasticity in the hippocampus of Disc1 mutant mice. Proceedings of the National Academy of Sciences of the United States of America. 2011;108(49):E1349-58. Epub 2011/11/04. doi: 10.1073/pnas.1114113108. PubMed PMID: 22049344; PubMed Central PMCID: PMCPmc3241761.

33. Kelly MP, Brandon NJ. Taking a bird's eye view on a mouse model review: A comparison of findings from mouse models targeting DISC1 or DISC1-interacting proteins. Future Neurology. 2011;6(5):661-77.

34. Grunewald E, Claxton C, Wang Q, Brandon NJ, Porteous DJ, Millar JK. Developmental profiling of DISC1, PDE4 and GSK3 in wild-type and Disc1 mutant mice. Society for Neuroscience Abstract Viewer and Itinerary Planner. 2011;41. PubMed PMID: BIOSIS:PREV201200097985.

35. Girgenti MJ, LoTurco JJ, Maher BJ. ZNF804a regulates transcription of the schizophrenia- associated genes PRSS16, COMT, NRG1, PDE4B, and DRD2. Neuropsychopharmacology. 2011;36:S337-S8.

36. Faucz FR, Bertollo De Alexandre R, Stratakis CA. Phosphodiesterases: Genes and their variants, inhibitors and potential therapeutic applications. Expert Review of Endocrinology and Metabolism. 2011;6(4):497-9.

37. Deng X, Takaki H, Wang L, Kuroki T, Nakahara T, Hashimoto K, et al. Positive association of phencyclidine-responsive genes, PDE4A and PLAT, with schizophrenia. American journal of medical genetics Part B, Neuropsychiatric genetics : the official publication of the International Society of Psychiatric Genetics. 2011;156b(7):850-8. Epub 2011/09/08. doi: 10.1002/ajmg.b.31233. PubMed PMID: 21898905.

38. Carlyle BC, Mackie S, Christie S, Millar JK, Porteous DJ. Co-ordinated action of DISC1, PDE4B and GSK3 beta in modulation of cAMP signalling. Molecular psychiatry. 2011;16(7):693-4. doi: 10.1038/mp.2011.17. PubMed PMID: WOS:000291973300005.

39. Braunewell KH, Dwary AD, Richter F, Trappe K, Zhao C, Giegling I, et al. Association of VSNL1 with schizophrenia, frontal cortical function, and biological significance for its gene product as a modulator of cAMP levels and neuronal morphology. Translational psychiatry. 2011;1:e22. Epub 2011/01/01. doi: 10.1038/tp.2011.20. PubMed PMID: 22832524; PubMed Central PMCID: PMCPmc3309514.

40. Tomppo L, Hennah W, Ekelund J, Lichtermann D, Veijola J, Miettunen J, et al. DISC1 conditioned genome-wide association study of psychosis proneness in a large finnish birth cohort. Schizophrenia research. 2010;117(2-3):454-5.

41. Souza RP, Remington G, Meltzer HY, Lieberman JA, Kennedy JL, Wong AH. Phosphodiesterase 4B genetic variants are not associated with antipsychotic-induced tardive dyskinesia. International clinical psychopharmacology. 2010;25(5):264-9. Epub 2010/05/04. doi: 10.1097/YIC.0b013e32833a5ff9. PubMed PMID: 20436352.

42. Newburn EN, Morita Y, Hyde TM, Kleinman JE, Lipska BK. Binding of human DISC1 isoforms to protein interactors. Society for Neuroscience Abstract Viewer and Itinerary Planner. 2010;40. PubMed PMID: BIOSIS:PREV201100574283.

43. Namba T, Kaibuchi K. Switching DISC1 Function in Neurogenesis: Dixdc1 Selects DISC1 Binding Partners. Developmental Cell. 2010;19(1):7-8. doi: 10.1016/j.devcel.2010.07.002. PubMed PMID: WOS:000280469100004.

44. Kelly MP, Logue SF, Brennan J, Day JP, Lakkaraju S, Jiang L, et al. Phosphodiesterase 11A in brain is enriched in ventral hippocampus and deletion causes psychiatric disease-related phenotypes. Proceedings of the National Academy of Sciences of the United States of America. 2010;107(18):8457-62. doi: 10.1073/pnas.1000730107. PubMed PMID: WOS:000277310400074.

45. Houslay MD. Underpinning compartmentalised cAMP signalling through targeted cAMP breakdown. Trends in biochemical sciences. 2010;35(2):91-100. Epub 2009/10/30. doi: 10.1016/j.tibs.2009.09.007. PubMed PMID: 19864144.

46. Guitart X, Numata S, Ye T, Hyde T, Weinberger D, Lipska B, et al. DNA methylation analysis of putative schizophrenia susceptibility genes in the human dorsolateral prefrontal cortex across the lifespan. Neuropsychopharmacology. 2010;35:S114.

47. Fatemi SH, Folsom TD, Reutiman TJ, Vazquez G. Phosphodiesterase signaling system is disrupted in the cerebella of subjects with schizophrenia, bipolar disorder, and major depression. Schizophrenia research. 2010;119(1-3):266-7. doi: 10.1016/j.schres.2010.02.1055. PubMed PMID: WOS:000280341700038.

48. Fatemi SH, Folsom TD, Reutiman TJ, Braun NN, Lavergne LG. Levels of phosphodiesterase 4A and 4B are altered by chronic treatment with psychotropic medications in rat frontal cortex. Synapse (New York, NY). 2010;64(7):550-5. Epub 2010/03/12. doi: 10.1002/syn.20762. PubMed PMID: 20222156.

49. Fatemi SH. Co-occurrence of neurodevelopmental genes in etiopathogenesis of autism and schizophrenia. Schizophrenia research. 2010;118(1-3):303-4. Epub 2010/02/16. doi: 10.1016/j.schres.2010.01.018. PubMed PMID: 20153953; PubMed Central PMCID: PMCPmc2856806.

50. Clapcote SJ, Lipina TV, Roder JC. Endophenotypes of Disc1 missense mutations in mice. European Neuropsychopharmacology. 2010;20:S200-S. PubMed PMID: WOS:000283687800107.

51. Burgin AB, Magnusson OT, Singh J, Witte P, Staker BL, Bjornsson JM, et al. Design of phosphodiesterase 4D (PDE4D) allosteric modulators for enhancing cognition with improved safety. Nature biotechnology. 2010;28(1):63-70. Epub 2009/12/29. doi: 10.1038/nbt.1598. PubMed PMID: 20037581.

52. Braunewell KH, Richter F, Dwary A, Pan W, Schoenrath K. The neuronal calcium sensor protein VILIP-1 as modulator of cAMP levels and differentiation in neurons - Implications for neurodevelopmental disturbances in schizophrenia. Society for Neuroscience Abstract Viewer and Itinerary Planner. 2010;40. PubMed PMID: BIOSIS:PREV201100534500.

53. Williams JM, Beck TF, Pearson DM, Proud MB, Cheung SW, Scott DA. A 1q42 Deletion Involving DISC1, DISC2, and TSNAX in an Autism Spectrum Disorder. American Journal of Medical Genetics Part A. 2009;149A(8):1758-62. doi: 10.1002/ajmg.a.32941. PubMed PMID: WOS:000268796000039.

54. Pedrosa E, Locker J, Lachman HM. Survey of schizophrenia and bipolar disorder candidate genes using chromatin immunoprecipitation and tiled microarrays (ChIP-chip). Journal of neurogenetics. 2009;23(3):341-52. Epub 2009/02/20. doi: 10.1080/01677060802669766. PubMed PMID: 19225952.

55. Numata S, Iga JI, Nakataki M, Tayoshi S, Taniguchi K, Sumitani S, et al. Gene expression and association analyses of the phosphodiesterase 4B (PDE4B) gene in major depressive disorder in the Japanese population. American Journal of Medical Genetics, Part B: Neuropsychiatric Genetics. 2009;150(4):527-34.

56. Mitchell KJ, Porteous DJ. GWAS for psychiatric disease: Is the framework built on a solid foundation. Molecular psychiatry. 2009;14(8):740-1.

57. Lipina TV, Kaidanovich-Beilin O, Patel S, Clapcote S, Liu F, Woodgett JR, et al. Genetic and pharmacological evidence for DISC1 interaction with GSK-3 in mice: Relevance to schizophrenia and depression. Society for Neuroscience Abstract Viewer and Itinerary Planner. 2009;39. PubMed PMID: BIOSIS:PREV201200029237.

58. Korth C. DISCopathies: brain disorders related to DISC1 dysfunction. Reviews in the neurosciences. 2009;20(5-6):321-30. Epub 2009/01/01. PubMed PMID: 20397618.

59. Kelly MP, Brandon NJ. Differential function of phosphodiesterase families in the brain: gaining insights through the use of genetically modified animals. Genetic Models of Schizophrenia. 2009;179:67-73. doi: 10.1016/s0079-6123(09)17908-6. PubMed PMID: WOS:000280617900009.

60. Hsu PC, Nwulia E, Akira S, Tamminga CA. Using bioinformatic tools. American Journal of Psychiatry. 2009;166(8):854.

61. Hennah W, Porteous D. The DISC1 pathway modulates expression of neurodevelopmental, synaptogenic and sensory perception genes. PloS one. 2009;4(3):e4906. Epub 2009/03/21. doi: 10.1371/journal.pone.0004906. PubMed PMID: 19300510; PubMed Central PMCID: PMCPmc2654149.

62. Drexhage H, Drexhage RC, Cohen D, Nolen W. RNA signature as diagnostic markers for schizophrenia. European Archives of Psychiatry and Clinical Neuroscience. 2009;259:S13.

63. Burdick KE, DeRosse P, Lencz T, Malhotra AK. Epistasis in the DISC1 Interactome: Effects on Risk for Schizophrenia. Biological psychiatry. 2009;65(8):203S-4S. PubMed PMID: WOS:000265144200646.

64. Bradshaw NJ, Ogawa F, Antolin-Fontes B, Chubb JE, Carlyle BC, Christie S, et al. DISC1, PDE4B, and NDE1 at the centrosome and synapse (vol 377, pg 1091, 2008). Biochemical and biophysical research communications. 2009;384(3):400-. doi: 10.1016/j.bbrc.2009.04.057. PubMed PMID: WOS:000266647400025.

65. Armstrong JD, Borkowska M, Dewar MA, Mackie S, Millar KJ, Porteous DJ. Targeted expression of DISC1 in the Drosophila brain disrupts courtship behaviour. Society for Neuroscience Abstract Viewer and Itinerary Planner. 2009;39. PubMed PMID: BIOSIS:PREV201200029248.

66. Wang Q, Jaaro-Peled H, Sawa A, Brandon NJ. How has DISC1 enabled drug discovery? Molecular and cellular neurosciences. 2008;37(2):187-95. Epub 2007/12/07. doi: 10.1016/j.mcn.2007.10.006. PubMed PMID: 18055216.

67. Soda T, Mao Y, Ge X, Frank CL, Tsai L. DISC1 regulates neural progenitor proliferation via modulation of GSK3 beta/beta-catenin signaling. Society for Neuroscience Abstract Viewer and Itinerary Planner. 2008;38. PubMed PMID: BIOSIS:PREV201200173125.

68. Siuciak JA, McCarthy SA, Chapin DS, Martin AN. Behavioral and neurochemical characterization of mice deficient in the phosphodiesterase-4B (PDE4B) enzyme. Psychopharmacology. 2008;197(1):115-26. Epub 2007/12/07. doi: 10.1007/s00213-007-1014-6. PubMed PMID: 18060387.

69. Siuciak JA. The Role of Phosphodiesterases in Schizophrenia. Cns Drugs. 2008;22(12):983-93. doi: 10.2165/0023210-200822120-00002. PubMed PMID: WOS:000261390900001.

70. Halene TB, Siegel SJ. Antipsychotic-like properties of phosphodiesterase 4 inhibitors: evaluation of 4-(3-butoxy-4-methoxybenzyl)-2-imidazolidinone (RO-20-1724) with auditory event-related potentials and prepulse inhibition of startle. The Journal of pharmacology and experimental therapeutics. 2008;326(1):230-9. Epub 2008/04/19. doi: 10.1124/jpet.108.138586. PubMed PMID: 18420599.

71. Fatemi SH, Reutiman TJ, Folsom TD, Lee S. Phosphodiesterase-4A expression is reduced in cerebella of patients with bipolar disorder. Psychiatric genetics. 2008;18(6):282-8. Epub 2008/11/20. doi: 10.1097/YPG.0b013e3283060fb8. PubMed PMID: 19018233.

72. Dlaboga D, Hajjhussein H, O'Donnell JM. Chronic haloperidol and clozapine produce different patterns of effects on phosphodiesterase-1B,-4B, and-10A expression in rat striatum. Neuropharmacology. 2008;54(4):745-54. doi: 10.1016/j.neuropharm.2007.12.002. PubMed PMID: WOS:000254231300012.

73. Camargo LM, Wang Q, Brandon NJ. What can we learn from the disrupted in schizophrenia 1 interactome: lessons for target identification and disease biology? Novartis Foundation symposium. 2008;289:208-16; discussion 16-21, 38-40. Epub 2008/05/24. PubMed PMID: 18497105.

74. Bradshaw NJ, Ogawa F, Antolin-Fontes B, Chubb JE, Carlyle BC, Christie S, et al. DISC1, PDE4B, and NDE1 at the centrosome and synapse. Biochemical and biophysical research communications. 2008;377(4):1091-6. Epub 2008/11/06. doi: 10.1016/j.bbrc.2008.10.120. PubMed PMID: 18983980.

75. Siuciak JA, Chapin DS, McCarthy SA, Martin AN. Antipsychotic profile of rolipram: efficacy in rats and reduced sensitivity in mice deficient in the phosphodiesterase-4B (PDE4B) enzyme. Psychopharmacology. 2007;192(3):415-24. Epub 2007/03/03. doi: 10.1007/s00213-007-0727-x. PubMed PMID: 17333137.

76. Polesskaya OO, Smith RF, Fryxell KJ. Chronic nicotine doses down-regulate PDE4 isoforms that are targets of antidepressants in adolescent female rats. Biological psychiatry. 2007;61(1):56-64. Epub 2006/07/04. doi: 10.1016/j.biopsych.2006.03.038. PubMed PMID: 16814262.

77. Murdoch H, Mackie S, Collins DM, Hill EV, Bolger GB, Klussmann E, et al. Isoform-selective susceptibility of DISC1/phosphodiesterase-4 complexes to dissociation by elevated intracellular cAMP levels. The Journal of neuroscience : the official journal of the Society for Neuroscience. 2007;27(35):9513-24. Epub 2007/08/31. doi: 10.1523/jneurosci.1493-07.2007. PubMed PMID: 17728464.

78. Millar KJ, Mackie S, Clapcote SJ, Murdoch H, Pickard BS, Christie S, et al. Disrupted in schizophrenia 1 and phosphodiesterase 4B: Towards an understanding of psychiatric illness. Journal of Physiology. 2007;584(2):401-5.

79. Mackie S, Millar JK, Porteous DJ. Role of DISC1 in neural development and schizophrenia. Current opinion in neurobiology. 2007;17(1):95-102. Epub 2007/01/30. doi: 10.1016/j.conb.2007.01.007. PubMed PMID: 17258902.

80. Kanes SJ, Tokarczyk J, Siegel SJ, Bilker W, Abel T, Kelly MP. Rolipram: a specific phosphodiesterase 4 inhibitor with potential antipsychotic activity. Neuroscience. 2007;144(1):239-46. Epub 2006/11/04. doi: 10.1016/j.neuroscience.2006.09.026. PubMed PMID: 17081698; PubMed Central PMCID: PMCPmc3313447.

81. Kakiuchi C, Ishiwata M, Nanko S, Kunugi H, Minabe Y, Nakamura K, et al. Association analysis of ATF4 and ATF5, genes for interacting-proteins of DISC1, in bipolar disorder. Neuroscience letters. 2007;417(3):316-21. Epub 2007/03/10. doi: 10.1016/j.neulet.2007.02.054. PubMed PMID: 17346882.

82. Clapcote SJ, Lipina TV, Millar JK, Mackie S, Christie S, Ogawa F, et al. Behavioral Phenotypes of Disc1 Missense Mutations in Mice. Neuron. 2007;54(3):387-402.

83. Cheung Y-F, Kan Z, Garrett-Engele P, Gall I, Murdoch H, Baillie GS, et al. PDE4B5, a novel, super-short, brain-specific cAMP phosphodiesterase-4 variant whose isoform-specifying N-terminal region is identical to that of cAMP phosphodiesterase-4D6 (PDE4D6). Journal of Pharmacology and Experimental Therapeutics. 2007;322(2):600-9. doi: 10.1124/jpet.107.122218. PubMed PMID: WOS:000248194600020.

84. Braun NN, Reutiman TJ, Lee S, Folsom TD, Fatemi SH. Expression of phosphodiesterase 4 is altered in the brains of subjects with autism. Neuroreport. 2007;18(17):1841-4. Epub 2007/12/20. doi: 10.1097/WNR.0b013e3282f16dca. PubMed PMID: 18090323.

85. Brandon NJ. Dissecting DISC1 function through protein-protein interactions. Biochemical Society transactions. 2007;35(Pt 5):1283-6. Epub 2007/10/25. doi: 10.1042/bst0351283. PubMed PMID: 17956330.

86. Shen S, St Clair D. Characterization of transgenic mice expressing a truncated disrupted-in-schizophrenia-1. American Journal of Medical Genetics Part B-Neuropsychiatric Genetics. 2006;141B(7):782-. PubMed PMID: WOS:000240877700434.

87. Sawamura N, Sawa A. Disrupted-in-Schizophrenia-1 (DISC1): A key susceptibility factor for major mental illnesses. 2006. p. 126-33.

88. Porteous DJ, Thomson P, Brandon NJ, Millar JK. The genetics and biology of DISC1 - An emerging role in psychosis and cognition. Biological psychiatry. 2006;60(2):123-31. doi: 10.1016/j.biopsych.2006.04.008. PubMed PMID: WOS:000239101300006.

89. Porteous DJ, Muir W, Blackwood D, Millar K, Pickard B. Genes and pathways through cytogenetics: The DISC1, PDE4B and GRIK4 paradigms. American Journal of Medical Genetics Part B-Neuropsychiatric Genetics. 2006;141B(7):689-. PubMed PMID: WOS:000240877700023.

90. Porteous DJ, Millar JK. Disrupted in schizophrenia 1: building brains and memories. Trends in molecular medicine. 2006;12(6):255-61. Epub 2006/05/09. doi: 10.1016/j.molmed.2006.04.009. PubMed PMID: 16679065.

91. Porteous DJ. DISC1: From genetics to function. Biological psychiatry. 2006;59(8):98S-9S. PubMed PMID: WOS:000236767300314.

92. Sawa A, Snyder SH. Two genes link two distinct psychoses. Science (New York, NY). 2005;310(5751):1128-9.

93. Sawa A, Snyder SH. Genetics. Two genes link two distinct psychoses. Science (New York, NY). 2005;310(5751):1128-9. Epub 2005/11/19. doi: 10.1126/science.1121114. PubMed PMID: 16293746.

94. Porteous D, Millar K, Pickard B, Mackie S, James R, Malloy P, et al. Disrupted in schizophrenia 1 and phosphodiesterase 4B are genetic factors in schizophrenia that interact to regulate cAMP signalling. American Journal of Medical Genetics Part B-Neuropsychiatric Genetics. 2005;138B(1):132-. PubMed PMID: WOS:000232357300478.

95. Pickard BS, Christoforou A, Thomson PA, Malloy MP, Evans KL, LeHellard S, et al. Beyond cytogenetics: Assessing the wider contribution of disrupted genes to schizophrenia and affective disorders through association studies. American Journal of Medical Genetics Part B-Neuropsychiatric Genetics. 2005;138B(1):51-2. PubMed PMID: WOS:000232357300182.

96. Millar JK, Pickard BS, Mackie S, James R, Christie S, Buchanan SR, et al. Genetics: DISC1 and PDE4B are interacting genetic factors in schizoprenia that regulate cAMP signaling. Science (New York, NY). 2005;310(5751):1187-91.

97. Millar JK, Pickard BS, Mackie S, James R, Christie S, Buchanan SR, et al. DISC1 and PDE4B are interacting genetic factors in schizophrenia that regulate cAMP signaling. Science (New York, NY). 2005;310(5751):1187-91. doi: 10.1126/science.1112915. PubMed PMID: WOS:000233437300046.

98. Pickard BS, Malloy MP, Birtley JR, LeHellard S, Hampson M, Ewald HL, et al. Cytogenetic abnormalities implicate glutamate neurotransmission, cAMP regulation and axonal guidance pathways in psychiatric illness. American Journal of Medical Genetics Part B-Neuropsychiatric Genetics. 2004;130B(1):72-. PubMed PMID: WOS:000223742600276.

99. Cherry JA, Thompson BE, Pho V. Diazepam and rolipram differentially inhibit cyclic AMP-specific phosphodiesterases PDE4A1 and PDE4B3 in the mouse. Biochimica Et Biophysica Acta-Gene Structure and Expression. 2001;1518(1-2):27-35. doi: 10.1016/s0167-4781(01)00164-6. PubMed PMID: WOS:000167822000004.

100. Pickard BS, Blackwood D, Porteous DJ, Muir WJ, Mors O, Ewald HL, inventors; Univ Edinburgh; Pickard B S; Blackwood D; Porteous D J; Muir W J; Mors O; Ewald H L, assignee. Use of a polynucleotide or a polypeptide for manufacturing a medicament for the treatment of schizophrenia and/or effective psychosis patent WO2003087408-A2; AU2003222965-A1; EP1492885-A2; JP2005522519-W; US2006088835-A1; WO2003087408-A3.

101. Konetzki I, Jakob F, Craan T, Hesslinger C, Ratcliffe P, Nardi A, inventors; Gruenenthal Gmbh, assignee. New substituted, condensed pyrimidine compounds useful for treating e.g. inflammatory diseases of joints, skin and eyes, gastrointestinal diseases, hyperplastic diseases, respiratory/lung diseases, cancers and metabolic diseases patent WO2014170020-A1.

102. Konetzki I, Jakob F, Craan T, Hesslinger C, inventors; Gruenenthal Gmbh, assignee. New pyrimidine compounds are phosphodiesterase 4B inhibitors, used to treat e.g. rheumatoid arthritis, gout, osteoarthritis, psoriasis, uveitis, Crohn's disease, lupus nephritis, asthma, pulmonary fibrosis, cough, glioma and type 2 diabetes patent WO2014117947-A1.

103. Hoffman CS, inventor; Boston College; Hoffman C S, assignee. Composition used in the treatment of e.g. penile erectile dysfunction, anxiety, depression, Alzheimer's disease, Parkinson's disease, asthma, pulmonary hypertension and stroke comprises phosphodiesterase inhibitor; and carrier patent WO2008130619-A2; WO2008130619-A9; WO2008130619-A3; US2010179158-A1; US2013344134-A1.

104. Edlin CD, Holman S, inventors; Glaxo Group Ltd, assignee. New quinoline compounds useful for treating e.g. asthma, emphysema, atopic dermatitis, allergic conjunctivitis, rheumatoid arthritis, multiple sclerosis, septic shock, and ulcerative colitis patent WO2007107499-A1.

105. Christensen SB, Holman S, Keeling SE, Sayani AP, inventors; Glaxo Group Ltd; Christensen S B; Holman S; Keeling S E; Sayani a P, assignee. New N-((1,6-diethyl-4-(tetrahydro-2H-pyran-4-ylamino)-1H-pyrazolo(3,4-b)pyri din-5-yl)methyl)-3-methyl-5-isoxazolecarboxamide useful for treating e.g. asthma, rheumatoid arthritis, urticaria, allergic conjunctivitis and depression patent WO2007036734-A1; EP1940836-A1; US2008255186-A1; JP2009510044-W.

106. Chappie TA, Verhoest PR, Patel NC, Hayward MM, inventors; Chappie T a; Verhoest P R; Patel N C; Hayward M M; Pfizer Inc, assignee. New azabenzimidazole compounds are phosphodiesterase 4B isoform binders useful for treating e.g. schizophrenia, depression, anxiety, Alzheimer's disease, multiple sclerosis, chronic obstructive pulmonary disease and inflammation patent US2014235612-A1; TW201443048-A.

**2. 7 studies excluded for not case-control studies.**

1. De Luca V, Zai C, de Souza R, Polsinelli G, Teo C, Shinkai T, et al. Admixture analysis of Age at Onset in Schizophrenia: Genetic Association Study of 45 candidate loci. Schizophrenia research. 2012;134(2-3):288-90.

2. Andreasen NC, Wilcox MA, Ho BC, Epping E, Ziebell S, Zeien E, et al. Statistical epistasis and progressive brain change in schizophrenia: an approach for examining the relationships between multiple genes. Molecular psychiatry. 2012;17(11):1093-102. Epub 2011/08/31. doi: 10.1038/mp.2011.108. PubMed PMID: 21876540; PubMed Central PMCID: PMCPmc3235542.

3. Semproni AR, Strick C, McDowell L, Mac-Dougal M, Mou K, Engle S, et al. Potential effect of PDE4B splice variant expression on PDE4 inhibitor activity in human, rat and murine cell based functional assays. Schizophrenia Bulletin. 2011;37:196.

4. Moens LN, De Rijk P, Reumers J, Van den Bossche MJ, Glassee W, De Zutter S, et al. Sequencing of DISC1 pathway genes reveals increased burden of rare missense variants in schizophrenia patients from a northern Swedish population. PloS one. 2011;6(8):e23450. Epub 2011/08/20. doi: 10.1371/journal.pone.0023450. PubMed PMID: 21853134; PubMed Central PMCID: PMCPmc3154939.

5. Wassink T, Ho BC, Ziebell S, Epping E, Andreasen N. A SNP array study identifies reelin and phosphodiesterase genes that influence cerebral cortical morphology in schizophrenia. Neuropsychopharmacology. 2010;35:S128.

6. Tomppo L, Hennah W, Lahermo P, Loukola A, Tuulio-Henriksson A, Suvisaari J, et al. Association between genes of Disrupted in schizophrenia 1 (DISC1) interactors and schizophrenia supports the role of the DISC1 pathway in the etiology of major mental illnesses. Biological psychiatry. 2009;65(12):1055-62. Epub 2009/03/03. doi: 10.1016/j.biopsych.2009.01.014. PubMed PMID: 19251251; PubMed Central PMCID: PMCPmc2696182.

7. Holliday EG, Nyholt DR, Tirupati S, John S, Ramachandran P, Ramamurti M, et al. Strong evidence for a novel schizophrenia risk locus on chromosome 1p31.1 in homogeneous pedigrees from Tamil Nadu, India. The American journal of psychiatry. 2009;166(2):206-15. Epub 2008/10/03. doi: 10.1176/appi.ajp.2008.08030442. PubMed PMID: 18829870.

**3. 2 studies excluded for not providing sufficient data, though we have tried to contact authors.**

1. Fatemi SH, King DP, Reutiman TJ, Folsom TD, Laurence JA, Lee S, et al. PDE4B polymorphisms and decreased PDE4B expression are associated with schizophrenia. Schizophrenia research. 2008;101(1-3):36-49. doi: 10.1016/j.schres.2008.01.029. PubMed PMID: WOS:000256212200005.

2. Pickard BS, Thomson PA, Christoforou A, Evans KL, Morris SW, Porteous DJ, et al. The PDE4B gene confers sex-specific protection against schizophrenia. Psychiatric genetics. 2007;17(3):129-33. doi: 10.1097/YPG.0b013e328014492b. PubMed PMID: WOS:000246512600001.

**4. 1 study written in Chinese excluded.**

1. Zhang L, Chen Y, He C, Ao L, Xing Y. Association study of schizophrenia and phosphodiesterase 4B gene polymorphism. Chinese Journal of Behavioral Medicine and Brain Science. 2010;19(5):429-31. PubMed PMID: CSCD:3955380.
